# Analysis of Modular Hub Genes and Therapeutic Targets across Stages of Non-Small Cell Lung Cancer Transcriptome

**DOI:** 10.3390/genes15101248

**Published:** 2024-09-25

**Authors:** Angeli Joy B. Barretto, Marco A. Orda, Po-wei Tsai, Lemmuel L. Tayo

**Affiliations:** 1 School of Chemical, Biological, and Materials Engineering and Sciences, Mapúa University, Manila City 1002, Philippines; ajbbarretto@mymail.mapua.edu.ph (A.J.B.B.); maorda@mymail.mapua.edu.ph (M.A.O.); 2 School of Graduate Studies, Mapúa University, Manila City 1002, Philippines; 3 Department of Food Science, National Taiwan Ocean University, Keelung 20224, Taiwan; powei@mail.ntou.edu.tw; 4 Department of Biology, School of Health Sciences, Mapúa University, Makati City 1203, Philippines

**Keywords:** non-small cell lung cancer (NSCLC), WGCNA, KEGG pathways, module preservation, drug repurposing, GW-5074, olomoucine, pinocembrin, citalopram, cell cycle regulation, protein binding, estrogen

## Abstract

Non-small cell lung cancer (NSCLC), representing 85% of lung cancer cases, is characterized by its heterogeneity and progression through distinct stages. This study applied Weighted Gene Co-expression Network Analysis (WGCNA) to explore the molecular mechanisms of NSCLC and identify potential therapeutic targets. Gene expression data from the GEO database were analyzed across four NSCLC stages (NSCLC1, NSCLC2, NSCLC3, and NSCLC4), with the NSCLC2 dataset selected as the reference for module preservation analysis. WGCNA identified eight highly preserved modules—Cyan, Yellow, Red, Dark Turquoise, Turquoise, White, Purple, and Royal Blue—across datasets, which were enriched in key pathways such as “Cell cycle” and “Pathways in cancer”, involving processes like cell division and inflammatory responses. Hub genes, including PLK1, CDK1, and EGFR, emerged as critical regulators of tumor proliferation and immune responses. Estrogen receptor ESR1 was also highlighted, correlating with improved survival outcomes, suggesting its potential as a prognostic marker. Signature-based drug repurposing analysis identified promising therapeutic candidates, including GW-5074, which inhibits RAF and disrupts the EGFR–RAS–RAF–MEK–ERK signaling cascade, and olomoucine, a CDK1 inhibitor. Additional candidates like pinocembrin, which reduces NSCLC cell invasion by modulating epithelial-mesenchymal transition, and citalopram, an SSRI with anti-carcinogenic properties, were also identified. These findings provide valuable insights into the molecular underpinnings of NSCLC and suggest new directions for therapeutic strategies through drug repurposing.

## 1. Introduction

NSCLC, or non-small cell lung cancer, accounts for over 85% of all cases of lung cancer and is one of the most common types [1,2]. This form of cancer is known for its heterogeneous nature, distinguishing it from small cell lung cancer (SCLC) by its typically slower progression and varied presentation. NSCLC is broadly classified into several subtypes based on histological characteristics and the anatomical locations of tumor development. The most common type, adenocarcinoma, often occurs in the lungs’ outer regions and arises from the glandular cells that border the alveoli [3]. Squamous cell carcinoma is typically found in the core regions of the lungs and develops from the squamous cells that border the airways [4]. Large cell carcinoma, while less common, can appear in any lung area and is noted for its rapid growth and tendency to spread quickly [5]. The progression of NSCLC is categorized into four distinct stages, each reflecting the extent of disease spread and influencing treatment decisions. Stage I NSCLC is categorized into two sub-stages, 1A and 1B, primarily based on tumor size. At this stage, the cancer remains localized to the lungs and has not yet invaded nearby lymph nodes or other body parts. Stage II is separated into IIA and IIB substages, with subcategories that consider tumor size, location, and lymph node involvement. While the tumor has not spread to distant organs, tumors in this stage may be larger than those in stage I and may have begun to damage neighboring lymph nodes. In stage III, which is subdivided into IIIA, IIIB, and IIIC, the cancer’s extent is assessed by tumor size, location, and the degree of lymph node spread, often involving the lymph nodes in the mediastinum, the region between the lungs. Stage IV represents the most advanced form of NSCLC, characterized by metastasis to the lining of the lungs or other distant sites in the body [6].

Non-small cell lung cancer (NSCLC) is treated using a range of therapies, including surgery, chemotherapy, radiation therapy, targeted therapy, and immunotherapy, modified to the stage of cancer, the patient’s overall health, and specific mutations found in the tumor [7]. Surgery is frequently the preferred therapy for early-stage NSCLC (stages I–IIIA), with a target of completely removing the tumor. Chemotherapy is widely utilized in advanced stages (IIIB and IV), either as neoadjuvant therapy to decrease tumors before surgery or as adjuvant therapy to eradicate remaining cancer cells after surgery. Cisplatin, carboplatin, paclitaxel, docetaxel, gemcitabine, and pemetrexed are common chemotherapy medicines frequently used in combination to enhance efficacy [8]. Radiation therapy can be used to treat patients who are unable to undergo surgery, as well as to provide palliative care to alleviate symptoms caused by tumor growth. Targeted therapy is very effective in advanced NSCLC, as it focuses on specific genetic abnormalities in cancer cells. Key targeted therapies include *RET* inhibitors like selpercatinib for *RET* rearrangements, ALK inhibitors like alectinib and crizotinib for *ALK* gene alterations, and *EGFR* inhibitors like osimertinib and erlotinib for *EGFR*-mutated cancers. *BRAF* inhibitors like dabrafenib and *MET* inhibitors like capmatinib are also utilized to treat particular mutations [9,10]. Overall, the therapy landscape for NSCLC is diverse, with several modalities suited to particular patient profiles and tumor features.

This study employs Weighted Gene Co-expression Network Analysis (WGCNA) to gain a better understanding of the molecular pathways underlying non-small cell lung cancer (NSCLC) and to discover possible treatment targets. Using gene expression data analysis, WGCNA is a bioinformatics technique that may be used to discover gene groups with similar expression patterns and build networks of co-expressed genes. Through the association of these gene groups with clinical characteristics, WGCNA offers insights into the molecular mechanisms underpinning distinct phases of the disease [11]. Four datasets of NSCLC—NSCLC1 (GSE19804), NSCLC2 (GSE43580), NSCLC3 (GSE101929), and NSCLC4 (GSE19804)—were examined using data from the Gene Expression Omnibus (GEO). Gene modules that are consistent across various datasets were identified with the help of WGCNA, and their biological relevance was determined using functional annotation and pathway enrichment analysis. To identify significant hub genes in each module, protein–protein interaction networks were also built. This could aid in the discovery of possible drug candidates [12].

With this bioinformatics approach, the study aims to shed light on the genetic differences and similarities across various stages of NSCLC. This understanding could reveal important insights that may contribute to enhancing current treatment strategies or developing new therapeutic options for NSCLC patients.

## 2. Materials and Methods

### 2.1. Dataset Acquisition

Microarray gene expression datasets for use in WGCNA analysis were obtained from the Gene Expression Omnibus (NCBI GEO) of the National Center for Biotechnology Information (https://www.ncbi.nlm.nih.gov/geo/, accessed on 25 July 2024). These datasets included primary lung tumor samples from patients with different stages of non-small cell lung cancer (NSCLC)—Stages 1 (NSCLC1), 2 (NSCLC2), 3 (NSCLC3), and 4 (NSCLC4)—and contained expression data. To ensure consistency and minimize variability due to differences in hybridization settings, probe sequences, and other platform-specific characteristics, only datasets generated with the GPL570-HG-U133 Plus 2 Affymetrix Human Genome U133 Plus 2.0 Array were selected [13]. A total of 74 samples were obtained. An overview of the information and sample distribution for each of the four NSCLC stages can be seen in Table 1.

The Bioconductor affy package (Bioconductor v3.18, http://www.bioconductor.org, accessed on 25 July 2024) provides an extensive toolset for analyzing Affymetrix microarray data [14]. It includes functions for reading Affymetrix CEL files, preprocessing the data, and performing various analyses. The robust multi-array average (RMA) method was used to process all screened raw data, which involved background correction, quantile normalization, and log-2 transformation. To visually inspect the resulting data for irregularities, sample clustering dendrograms and a boxplot of expression values were created [15]. After removing control probes and applying variance filtering, the expression datasets were subset to include only common probes, focusing on lung tissue tumor expression samples while eliminating non-biological variation. The datasets were further refined by retaining only similar rows and removing data entries lacking gene symbols. Probe IDs were converted to gene symbols using the hgu133plus2.db database and the AnnotationDbi function. Finally, only probes present in all datasets were used, and samples without values after log-2 transformation were removed using the ‘goodSamplesGenesMs’ function from the WGCNA R package (R version 4.4.0).

### 2.2. Weighted Gene Co-Expression Network Analysis (WGCNA)

#### 2.2.1. Estimating a Scale-Free Network

The ‘pickSoftThreshold’ function from the WGCNA R package was employed to assess the adherence of network data to a scale-free topology. This function generated a plot that evaluated the fit of the data to a scale-free topology model across a range of power levels (1 to 20). In a scale-free network, characterized by a power-law distribution, a minority of nodes (genes) have a large number of connections, while most have relatively few connections. The optimal power level (β) was identified as the lowest value at which the network begins to approximate a scale-free topology. This was determined by locating the point where further increases in power did not significantly improve the fit of the data to the scale-free model [16]. To validate the scale-free nature of the network, the relationship between the logarithm of the number of connections (log connectivity) and the logarithm of the probability of those connections (log connectivity probability) was analyzed. A linear trend in this plot would suggest that the network follows a scale-free distribution [17]. The plot of scale-free topology fit versus soft-thresholding power was examined to identify the point where the fit plateaus or reaches a high value. Additionally, the values of soft connectivity (k) were plotted to confirm the appropriateness of the selected power level (β).

#### 2.2.2. Module Identification and Network Construction Using TOM

Pearson’s correlation was used to calculate the strength of the correlation between genes. Using the network type ‘signed’, this analysis considers the direction of the correlation (positive or negative). The Topological Overlap Measure (TOM) was used to evaluate the similarity of connection patterns between gene pairs within the network. Adjacency matrices were created to represent the strength of correlations between genes, which were adjusted using the chosen power level to emphasize strong correlations and downweight weak ones. The flashClust function was used to group genes into clusters based on their similarity, and the hclust function was employed to cut the dendrograms into distinct clusters (modules) of highly correlated genes. Hierarchical clustering was performed using the ‘average’ method, based on the distance matrix of the expression profiles. Different values (0 to 3) were tested using the cutreeHybrid function to determine the optimal level for splitting the dendrogram branches [18]. Consistent clusters over a range of deep split parameter values indicate successful detection of relevant gene modules.

#### 2.2.3. Module Preservation Analysis

Gene modules from NSCLC1, NSCLC2, NSCLC3, and NSCLC4 were analyzed using the modulePreservation function from the WGCNA R package, configured with a ‘signed’ network type to account for the direction of gene correlations. To evaluate the stability and significance of module preservation, 100 random permutations were performed, and only modules containing at least 30 genes were included in the analysis. Module membership (kME), quantifying the correlation between each gene and its module’s overall expression profile, was calculated using the moduleEigengenes function, which generates a representative expression profile (eigengene) for each module. kME values for each gene were then computed based on their correlation with the module eigengene. This approach evaluates whether gene connection patterns observed under specific conditions, such as different disease states, are consistent across other conditions [19]. The stability and relevance of these gene modules were assessed by identifying key genes with strong correlations to the overall module expression profile.

### 2.3. Enrichment Analysis and KEGG Pathway

The Database for Annotation, Visualization, and Integrated Discovery (DAVID) was used for functional annotation clustering to assess the relevance of gene modules. The analysis incorporated three Gene Ontology (GO) categories: Biological Processes (BP), describing the roles and processes the genes are involved in; Cellular Components (CC), indicating the cellular locations of the genes or their products; and Molecular Functions (MF), detailing the activities of the gene products, such as binding or catalytic activity. The classification stringency was set to medium, grouping genes based on their functions with moderate strictness. Only GO terms with enrichment scores higher than 1.3 and an adjusted p-value of less than 0.05 were considered significant. Additionally, pathway enrichment analysis was conducted using the Kyoto Encyclopedia of Genes and Genomes (KEGG) database to identify pathways in which the genes are involved. Significant KEGG terms were pathways that were significantly enriched and clustered with the selected GO terms. These significant KEGG and GO terms helped to understand the broader biological functions and roles of the gene modules, providing insights into the activity locations of the genes, their functions, and the biological processes they participate in.

### 2.4. Protein–Protein Interaction and Hub Genes Identification

Protein–protein interaction (PPI) networks were generated for each of the highly preserved modules using Cytoscape v3.10.2. The networks were constructed by importing data from the public database STRING using the protein query as the data source, with a minimum cutoff score of 0.7 to ensure high confidence. After generating these networks in Cytoscape, the CytoHubba tool (v0.1.) was used to identify hub genes by evaluating their degree of centrality, which indicates the number of interactions a gene has in the network and its importance relative to other genes [20,21,22]. Hub genes were defined as the top five genes in each module with the highest number of interactions.

### 2.5. Signature-Based Drug Repurposing 

The top 10 hub genes were first categorized as ‘upregulated’ or ‘downregulated’ using GEO2R (https://www.ncbi.nlm.nih.gov/geo/geo2r/, accessed on 25 July 2024) based on degree centrality. Following this classification, transcription profiles from the Molecular Signatures Database (MSigDB) and Connectivity Map (CMap) were used to identify drug candidates for repurposing via the Drug Repurposing Encyclopedia (DRE) [23,24]. The lists of upregulated and downregulated genes were submitted for drug repurposing analysis using molecular signature screening. Only drugs with clearly indicated mechanisms of action and false discovery rates (FDR) below 0.05 were included in the final analysis, while experimental drugs without specified mechanisms of action were excluded [15].

### 2.6. Language Refinement with AI Tools

In this study, AI tools, specifically OpenAI’s ChatGPT (GPT-4o mini) and Grammarly (v1.2.0.0), were used to assist in the revision of the manuscript for grammar correction and sentence construction. These tools were employed to improve the clarity and readability of the text after experimental content had been fully developed. No AI tools were used for data analysis, interpretation of results, or shaping conclusions. No research data or images provided here were generated or created with the assistance of any AI tools.

## 3. Results

### 3.1. Weighted Gene Co-Expression Network Analysis

#### 3.1.1. Data Pre-Processing and Scale-Free Network Estimation

**Figure 1:**
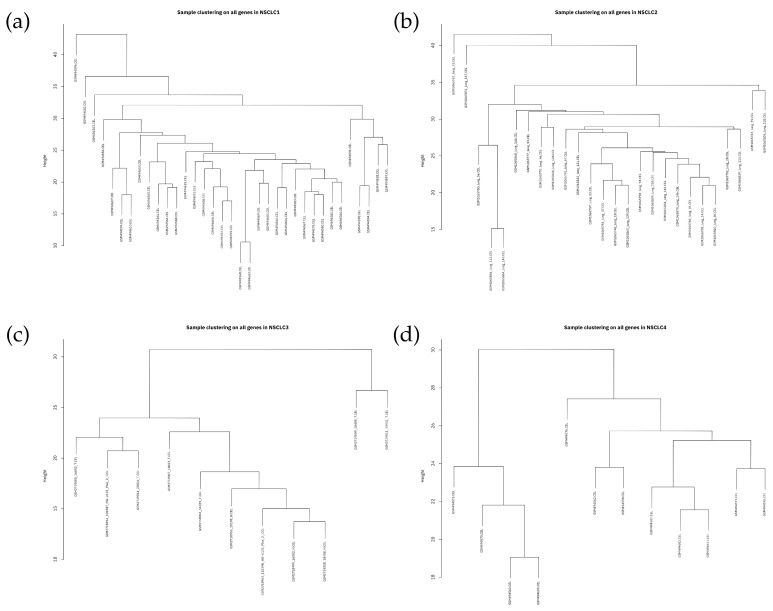
Sample clustering dendrograms for (**a**) NSCLC1, (**b**) NSCLC2, (**c**) NSCLC3, (**d**) NSCLC4.

**Figure 2:**
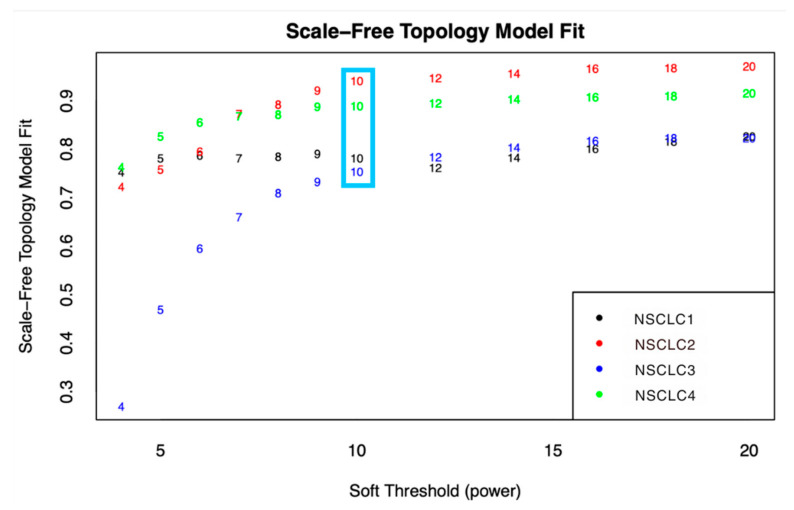
The soft-thresholding power estimated from the four datasets was found using the network index plot and Scale-Free Topology Model Fit. The power at which the index began to plateau is indicated by the blue box.

After preparing the data and filtering the genes, a total of 27,872 genes remained for further processing. The sample clustering dendrograms for each dataset, shown in Figure 1, revealed no clear outliers. Figure 2 depicts the variation of the scale-free topology fit index versus the soft-thresholding power (β) ranging from 1 to 20, illustrating the Scale-Free Topology Model Fit. The model starts to stabilize and form a linear trend when the power (β) reaches 10. Powers greater than 10 do not significantly improve the fit, as a power of 10 already provides a good scale-free structure. To construct biologically significant gene co-expression networks with minimal noise and information loss, it is crucial to approximate the ideal soft-thresholding power in WGCNA [25,26]. Generally, the soft-thresholding power decreases rapidly with increasing values, so selecting the lowest power that meets the scale-free topology standards is beneficial.

**Figure 3:**
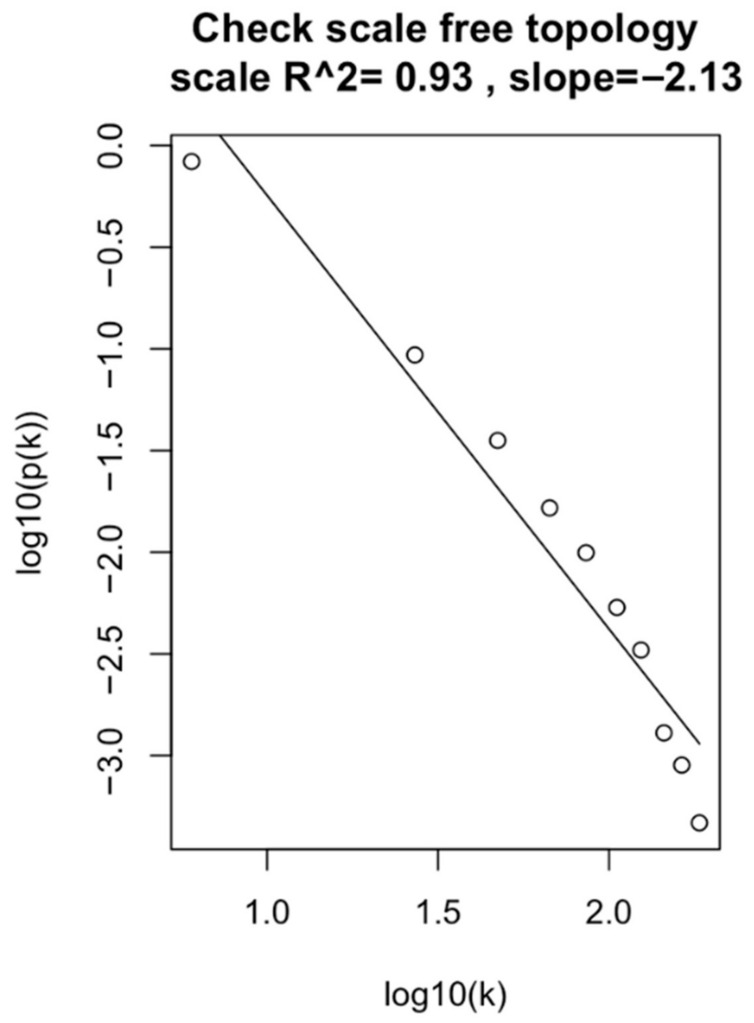
The log-log plot of the approximated linear relationship for the NSCLC2 dataset at β = 10.

Figure 3 shows the log-log plot of the NSCLC2 dataset, illustrating the frequency of different connectivity values with a power value of 10. Among all datasets, the NSCLC2 dataset demonstrated the best results with an R^2^ value of 0.93. This high R^2^ value indicates strong gene connections and reliable results with clear patterns. Consequently, the NSCLC2 dataset was selected as the reference dataset for further analysis.

#### 3.1.2. Identification of Modules Using TOM-Based Network Construction

**Figure 4:**
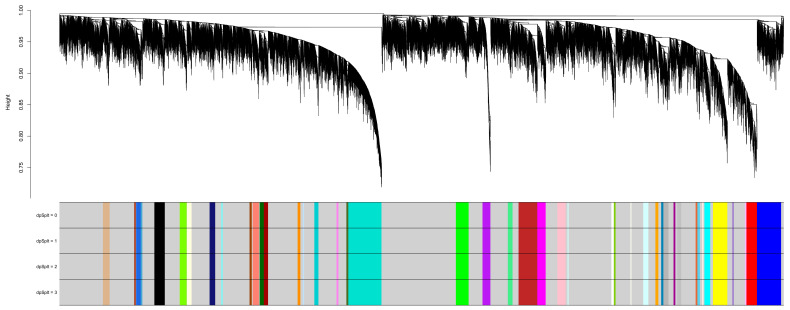
Dendrogram demonstrating module split sensitivity and gene clustering on TOM-based dissimilarity in NSCLC2. The different colored sections show the detected gene co-expression modules that match the area of the dendrogram.

In TOM-based network construction and module identification, it is crucial to identify a reference dataset and project the module eigengenes of other datasets onto it. Module eigengenes, calculated as the first principal component of the expression data for all genes within a module, serve as summary statistics capturing the overall expression level of the module. Standardizing module definitions and improving biological interpretation are essential for facilitating cross-dataset analysis and meta-analyses [27]. By projecting the module eigengenes of different datasets onto a reference dataset (in this case, NSCLC2), cross-dataset comparisons and biological interpretations are enhanced. Consequently, NSCLC2 was chosen as the reference dataset for meta-analysis, and the identified modules are depicted in Figure 4.

In the NSCLC2 dataset analyzed using WGCNA, a total of 48 gene co-expression modules were discovered and categorized by color. The largest module, “grey”, contained 2500 genes, followed by “turquoise” with 1023 genes and “blue” with 744 genes. The “yellow” module had 440 genes, while the “brown” module included 575 genes. Other notable modules were “red” (321 genes), “black” (320 genes), and “green” (393 genes). Smaller modules, such as “floralwhite” (30 genes), “lightsteelblue1” (35 genes), and “ivory” (31 genes), were also identified. The diversity in module sizes reflects the impact of sample size and clustering resolution on network robustness. These modules represent groups of related genes with coordinated expression patterns across NSCLC stages, providing insights into the molecular processes and regulatory networks involved. Additionally, associating module eigengenes with external traits or clinical outcomes helps infer the biological significance of the modules and enhances the overall interpretation of the findings [28].

### 3.2. Module Preservation Analysis

**Figure 5:**
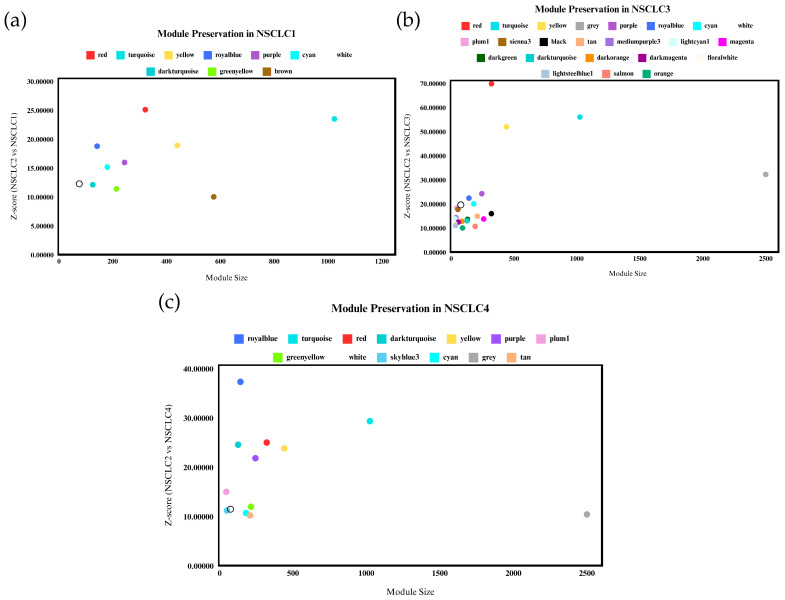
Gene co-expression modules from the NSCLC2 network’s (**a**) NSCLC1, (**b**) NSCLC3, and (**c**) NSCLC4 datasets were subjected to a module preservation analysis. Highly maintained modules are those with a Z-score greater than or equal to 10.

The preservation of gene co-expression network modules in non-small cell lung cancer stage 2 (NSCLC2) was evaluated across different stages of NSCLC (NSCLC1, NSCLC3, NSCLC4) using the highest Z-summary score for better preservation assessment. Only modules with a Z-score ≥ 10 across all datasets were considered significant [29]. Figure 5 illustrates the preservation of different modules across stages. Minor variations were observed between the preserved modules across datasets. Eight modules with high Z-scores were identified across all datasets: Red, Turquoise, Yellow, Royal Blue, Purple, Cyan, White, and Dark Turquoise. These modules are preserved across stages, indicating shared networks among different NSCLC stages. NSCLC3 exhibited the most significantly preserved modules compared to other stages, likely due to its transitional nature, where the cancer is more aggressive and may metastasize to nearby organs, potentially leading to NSCLC4 [24]. Eigengene-based connectivity (kME) was used to assess module membership and identify hub genes within co-expression modules. Genes with high kME values exhibit strong correlations with other genes within the same module and are ranked based on their connectivity. Top genes with the highest kME scores from each module were selected as promising biomarkers and therapeutic targets due to their high connectivity and association with disease progression and poor survival in NSCLC [27].

### 3.3. Gene Ontology (GO) Terms and KEGG Pathway Analysis

**Figure 6:**
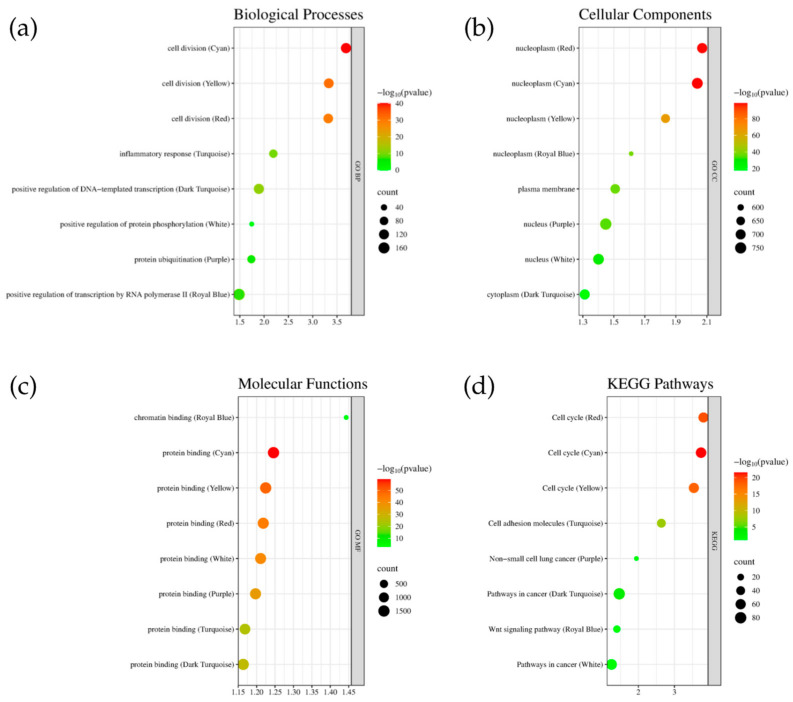
Top enriched terms for the Cyan, Yellow, Red, Turquoise, Dark Turquoise, White, Purple, and Royal Blue modules in terms of (**a**) biological processes, (**b**) cellular components, (**c**) molecular functions, and (**d**) KEGG pathways.

Figure 6 presents the GO terms and KEGG pathway analysis for the highly preserved genes in each module. The enrichment scores indicate significant connections to NSCLC progression. Specifically, the Cyan, Yellow, and Red modules are involved in cell division processes. The cellular components analysis shows that the Red, Cyan, and Yellow modules are all active in the nucleoplasm. Protein binding is prominent across all modules except Royal Blue. In the KEGG pathway analysis, the cell cycle is notably involved in the Red, Cyan, and Yellow modules. Additionally, the Purple pathway shows significant involvement in NSCLC, underscoring its relevance. The Dark Turquoise and White modules are associated with general ‘Pathways in Cancer’, with NSCLC being a specific type of lung cancer. Functional annotations were grouped using the DAVID webserver based on the top module.

### 3.4. Protein–Protein Interaction Network and Key Hub Genes

**Figure 7:**
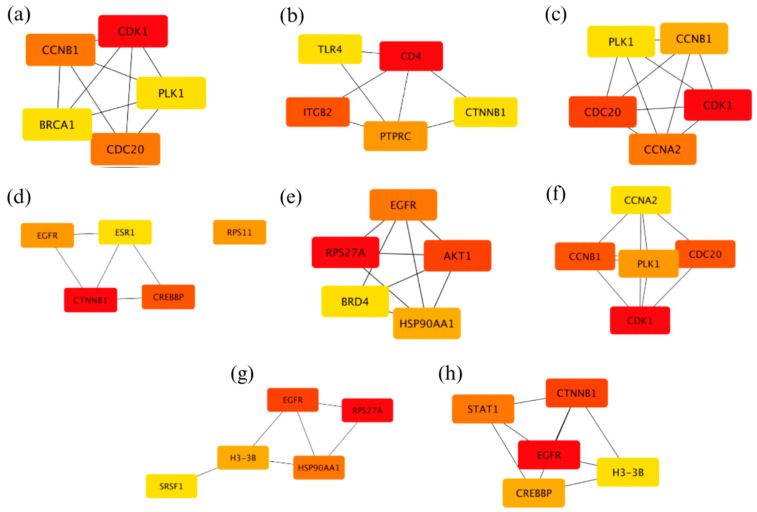
Top five hub genes based on the PPI network of the modules (**a**) Red, (**b**) Turquoise, (**c**) Yellow, (**d**) Royal Blue, (**e**) Purple, (**f**) Cyan, (**g**) White, and (**h**) Dark Turquoise.

Using Cytoscape v3.10.2, the identified modules in gene co-expression networks were cross-referenced with PPI networks to validate and enhance the understanding of the underlying biological processes. A cutoff score of 0.7 was applied to ensure a high confidence level in the interactions [30]. The top five hub genes for each module were identified based on degree ranking using the Cytoscape CytoHubba plugin. Figure 7 displays these top five hub genes, with their rankings indicated by color intensity, where the brightest red represents the highest-ranking gene. After identifying the hub genes in the PPI network, the next step involved integrating these genes into broader signaling and regulatory networks. This integration helps elucidate their interactions with other pathways and their roles in the complex biological systems underlying NSCLC.

### 3.5. Signature-Based Drug Repurposing

Drugs that produce gene expression profiles inversely matched to the NSCLC hub gene signature were identified using DRE. These drugs hold potential for repurposing as they may counteract the distinctive expression patterns of hub genes associated with NSCLC. Table 2 presents a selection of the most promising drug candidates along with their relevant mechanisms of action. The false discovery rate (FDR) is a statistical metric that estimates the percentage of false positives among positive results, with lower FDR values indicating more reliable drug candidates [31]. In gene expression and drug development, a greater negative tau value often signifies a more pronounced and effective alteration in gene expression. However, tau values can have varying implications depending on the context [32]. The top-ranked drug candidates for upregulated expression include L-745870, pentolinium, GW-5074, pinocembrin, and bisphenol-A. For downregulated hub genes, the most promising therapeutic candidates are clopidogrel, RX-821002, FR-122047, olomoucine, and citalopram.

## 4. Discussion

### 4.1. Gene Co-Expression Modules across the Datasets

**Figure A1:**
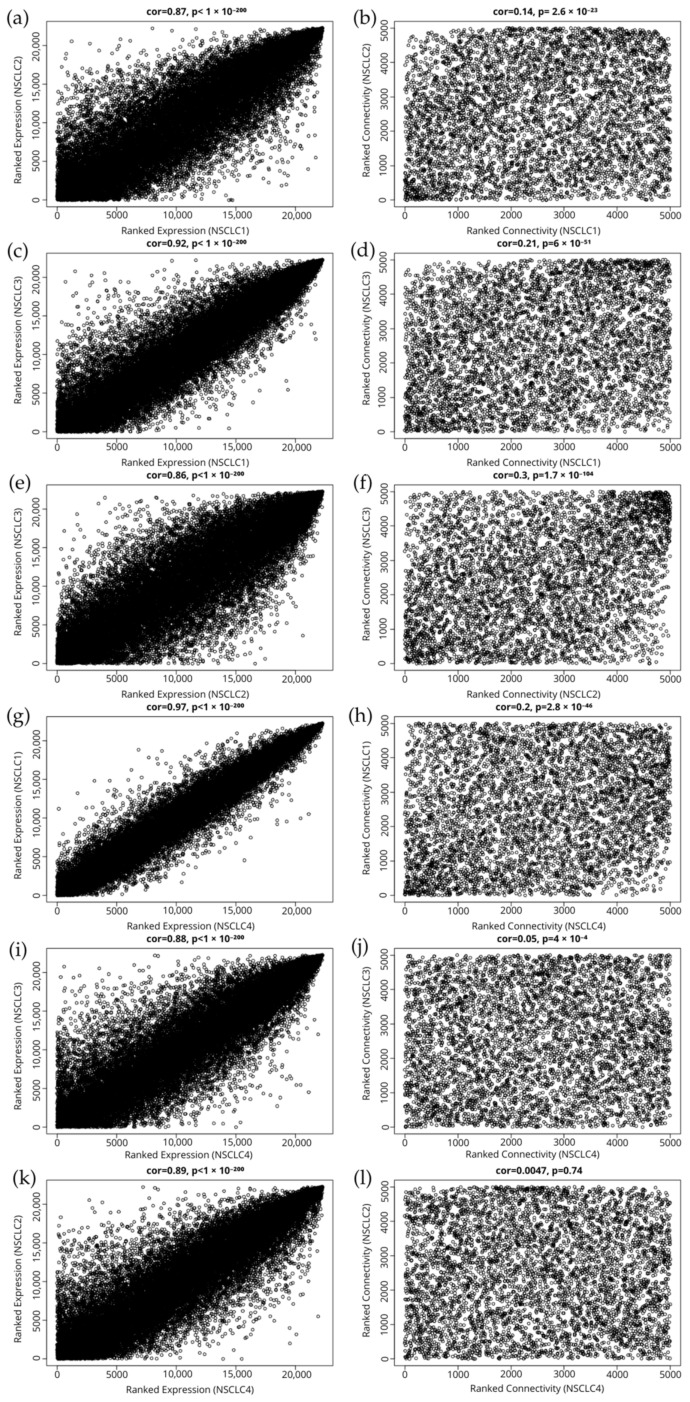
Ranked expression plots and ranked connectivity plots for dataset comparison (**a**,**b**) NSCLC1 vs. NSCLC2; (**c**,**d**) NSCLC1 vs. NSCLC3; (**e**,**f**) NSCLC2 vs. NSCLC3; (**g**,**h**) NSCLC4 vs. NSCLC1; (**i**,**j**) NSCLC4 vs. NSCLC3; and (**k**,**l**) NSCLC4 vs. NSCLC2.

Weighted Gene Co-expression Network Analysis (WGCNA) is a powerful bioinformatics method for analyzing gene expression data, particularly in diseases such as non-small cell lung cancer (NSCLC). By identifying gene modules correlated with disease symptoms, WGCNA provides valuable insights into disease progression and potential treatment targets [33,34]. NSCLC is the most common type of lung cancer, accounting for approximately 85% of all lung cancer diagnoses [35]. The disease’s progression is often influenced by genetic abnormalities and changes in gene expression [36]. WGCNA constructs co-expression networks that reveal gene interactions and their impact on tumor activity. It also identifies pathways involved in disease networks by analyzing the interactions of highly preserved genes. This methodology supports the application of WGCNA to explore the disease network across various NSCLC stages, represented by the NSCLC1, NSCLC2, NSCLC3, and NSCLC4 datasets. NSCLC3, a transitional stage where the cancer is more aggressive and may spread to other organs [35], showed a higher number of preserved modules compared to other stages. This is reflected in Figure A1, which indicates a significant correlation (0.86) between NSCLC2 and NSCLC3, suggesting that despite representing different stages, their molecular characteristics overlap significantly. Enrichment analysis using DAVID revealed that the highly preserved modules Cyan, Yellow, and Red are predominantly involved in cell division, while the Turquoise module is associated with inflammatory responses (Figure 6a). The link between the Cyan, Yellow, and Red modules and cell division is particularly relevant for NSCLC, given the disease’s association with dysregulation of cell cycle processes. Research has shown that differentially expressed genes (DEGs) in NSCLC are significantly involved in various aspects of the cell cycle, including mitotic nuclear division and regulation [37,38]. The cellular component analysis (Figure 6b) highlights that modules like Dark Turquoise are primarily associated with nucleoplasm and cytoplasm. Alterations in nuclear body composition and function can disrupt gene expression regulation. For example, interactions between nuclear bodies and chromatin are crucial for maintaining transcriptional states. Highly transcribed genes often associate with proteins of the nuclear pore complex, facilitating RNA and protein transport between the nucleus and cytoplasm, which can impact cancer progression [39]. The cellular components of the nucleoplasm, particularly nuclear bodies, are integral to gene regulation and cellular responses, with significant implications for the pathophysiology of NSCLC. In general, proteins exhibit a wide range of binding capabilities, which are crucial for various biological functions. In terms of molecular function, all identified modules (Figure 6c) show significant involvement in protein binding. This interaction is essential for numerous biological processes, including signal transduction, enzymatic activity, and structural integrity within cells [40]. In NSCLC, protein binding is critical for tumor progression and metastasis. For example, proteins involved in *EGFR* signaling pathways are crucial for NSCLC development and maintenance. Ligand binding to *EGFR* can activate downstream signaling pathways that promote cell proliferation and survival, contributing to cancer progression [41]. A key limitation of this study is the small sample sizes for the NSCLC3 and NSCLC4 stages, which include only 11 and 12 samples, respectively. These limited numbers may affect the general application of the findings, as smaller samples can reduce statistical power and may not adequately represent the diversity within these stages. This limitation could impact the reliability of the identified gene modules and potential therapeutic targets, particularly regarding their applicability to a wider range of patients. While the results provide important insights into the molecular mechanisms of NSCLC progression, it is essential to exercise caution when applying these findings to the broader NSCLC population. To strengthen the conclusions drawn from this study, future research should focus on larger and more balanced sample sizes across all NSCLC stages. This approach will help validate and refine the findings, ultimately contributing to more effective treatment strategies for NSCLC.

**Figure 8:**
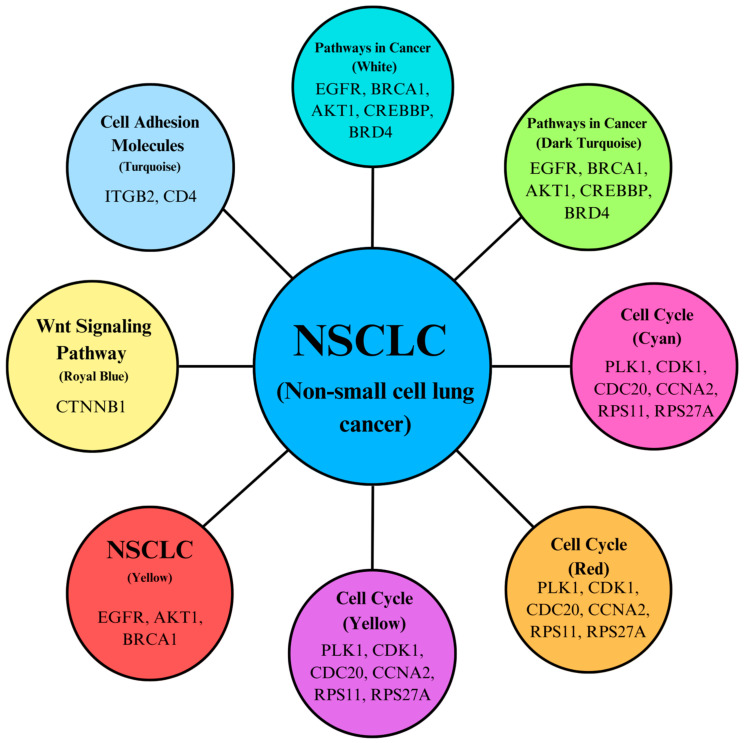
KEGG pathways interconnectivity based on the functions of key hub genes.

KEGG pathway analysis, as shown in Figure 8, highlights the hub genes associated with each pathway within the modules. The significant enrichment of the “Cell cycle” pathway underscores the dysregulation of cell cycle control mechanisms in NSCLC, which can lead to uncontrolled cell division. A study emphasizes the importance of cell cycle regulators, such as *CDK1* and cyclins, in NSCLC, indicating that targeting these regulators can inhibit tumor growth and induce apoptosis in NSCLC cells [42]. The “Pathways in cancer” enrichment reflects the involvement of multiple signaling pathways in NSCLC tumorigenesis. Research by Cui et al. discusses the role of various oncogenes and tumor suppressor genes in NSCLC, including mutations in *EGFR*, *K-RAS*, and *p53*, which are critical in cancer pathways. These genetic alterations lead to uncontrolled cell proliferation and survival, highlighting their significance in NSCLC progression [43]. The “non-small cell lung cancer” pathway specifically addresses the molecular mechanisms underlying NSCLC, including key genetic alterations. The KEGG pathway analysis provides a comprehensive view of signaling pathways involved in NSCLC, emphasizing the roles of *K-RAS* mutations and *EGFR* overexpression in promoting cell proliferation and survival. This reinforces the relevance of these pathways in understanding NSCLC biology. Additionally, the “Cell adhesion molecules” pathway highlights the importance of cell–cell and cell–matrix interactions in NSCLC, which can impact tumor invasion and metastasis [42]. The study examines how alterations in cell adhesion molecules contribute to NSCLC metastasis, suggesting that targeting these interactions may help inhibit tumor spread and improve patient outcomes. This analysis suggests that there may be interactions, cross-talk, or amplification within these pathways that contribute to NSCLC progression.

**Figure 9:**
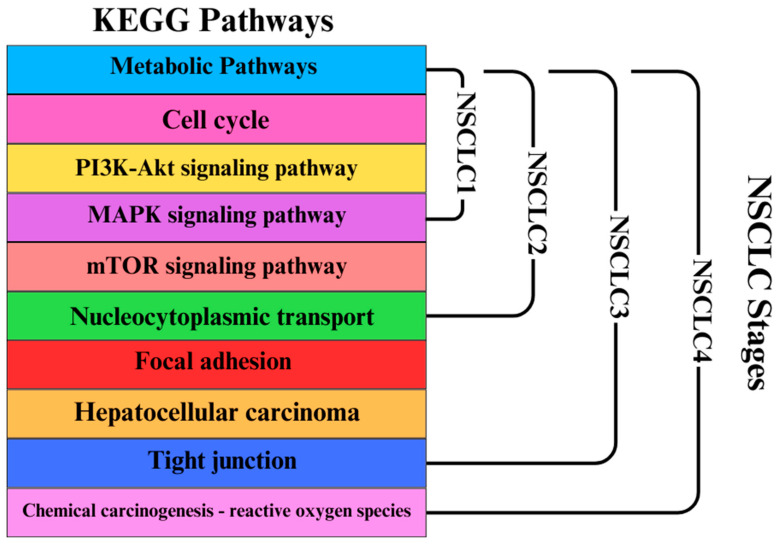
Modules in KEGG pathways with increased preservation for each NSCLC stage (NSCLC1, NSCLC2, NSCLC3, NSCLC4).

Additionally, the increased preservation of modules per stage is demonstrated by Figure 9, which displays the top KEGG pathways per module that could potentially act as significant point in the progression of NSCLC. Modules that reached the threshold of z ≥ 10 per stage were all considered in this figure. A study published in April 2024 demonstrated that targeted inhibition of the *PI3K*/*AKT*/*mTOR* pathway using specific compounds led to cell cycle arrest, apoptosis, and autophagy in NSCLC cells. This suggests that *mTOR* is a viable target for therapeutic intervention in NSCLC, providing a rationale for developing *mTOR* inhibitors as part of treatment strategies [44]. Studies have shown that alterations in nucleocytoplasmic transport mechanisms can lead to the aberrant localization of oncogenes and tumor suppressor proteins. For example, mutations in genes like *p53* can disrupt normal transport processes, leading to enhanced cell proliferation and survival, which are hallmarks of NSCLC [45]. Research has identified specific genes within the focal adhesion pathway that are upregulated in NSCLC. A study found that genes such as *VWF*, *CAV1*, and *ITGA8* were significantly enriched in the focal adhesion pathway and associated with poor prognosis in NSCLC patients [43]. This is also why it is present in the late stages of NSCLC, as shown in Figure 9. These findings suggest that focal adhesion signaling may play a critical role in the aggressiveness of NSCLC tumors. According to research, the *PI3K*/*Akt*/*mTOR* signaling pathway—which is essential for cell growth and survival—is one of the oncogenic pathways shared by both HCC and NSCLC. In both cancers, deviations in these pathways might result in the growth of tumors. According to a study, NSCLC and HCC pathway-related genes were found to be highly expressed in each other, indicating a possible molecular connection between the two cancers [43]. Tight junctions are crucial for maintaining epithelial integrity and regulating paracellular permeability. In NSCLC, alterations in tight junction proteins can lead to increased invasiveness and metastasis. A study indicated that the dysregulation of tight junctions contributes to the epithelial–mesenchymal transition (EMT), a process associated with enhanced migratory and invasive properties of cancer cells [46]. The pathway details how chemical carcinogens can induce oxidative stress, leading to DNA damage and mutations, which are critical in the initiation and progression of lung cancer. A study by Henkler et al. (2010) discusses the role of oxidative stress in carcinogenesis induced by metals and xenobiotics, emphasizing the importance of ROS in cancer development, including lung cancer [47]. These findings could lead to new research studies in NSCLC, including computational modeling, laboratory experiments, and clinical studies.

### 4.2. Module Hub Genes and Their Functions

A number of hub genes play crucial roles in the pathophysiology and progression of non-small cell lung cancer (NSCLC), affecting various cellular processes. These genes can be categorized based on their functions in ribosomal protein synthesis, transcription, immune response, signal transduction, cell cycle regulation, and DNA damage repair. The hub genes associated with each module and their functions are listed in Table A1, derived from the GeneCards database (www.genecards.org, accessed 29 July 2024) [48].

#### 4.2.1. Cell Cycle Regulation and Proliferation

The analysis of gene co-expression networks and module preservation in NSCLC datasets unveiled crucial insights into the disease’s pathophysiology. Weighted Gene Co-expression Network Analysis (WGCNA) identified several significant gene modules across different stages of NSCLC, with prominent modules related to cell cycle regulation and mitotic progression. These findings were further validated by KEGG pathway analysis, which highlighted the “Cell cycle” pathway as significantly enriched in modules such as Red, Cyan, and Yellow (Figure 8).

Polo-like kinase 1 (*PLK1*), identified as a hub gene in the context of mitotic regulation, was found to be a key player in the modules associated with cell cycle processes. Elevated *PLK1* expression has been associated with poor prognosis and enhanced cell proliferation in NSCLC [49]. This is supported by the results observed in the module preservation analysis, where modules associated with cell division and mitosis showed high preservation across different stages of NSCLC (Figure 4 and Figure 5). Elevated *PLK1* levels align with the observed enrichment in the “Cell cycle” pathway, reinforcing its role as a critical factor in NSCLC progression and a potential therapeutic target [50,51]. Targeting *PLK1* with inhibitors, such as volasertib, has shown potential in improving NSCLC cells’ radiosensitivity [52,53]. Cyclin-dependent kinase 1 (*CDK1*) and Cell Division Cycle 20 (*CDC20*) were also identified as hub genes within modules associated with mitotic progression. Their dysregulation, particularly in the context of the G2/M transition, aligns with the results from the functional annotation clustering and pathway enrichment analyses. Modules Cyan, Yellow, and Red, which were highly preserved and enriched in cell cycle processes, underscore the critical role of these regulators in uncontrolled proliferation. The significant enrichment of the “Cell cycle” pathway across these modules highlights the importance of *CDK1* and *CDC20* in NSCLC, as their dysregulation contributes to tumor aggressiveness and resistance to treatment [54]. Cyclin A2 (*CCNA2*), involved in regulating the S phase and G2/M transition, was similarly identified as a crucial hub gene within the modules associated with cell cycle processes. The correlation between *CCNA2*’s role in cell cycle regulation and the observed enrichment in the “Cell cycle” pathway further supports the relevance of these findings in understanding NSCLC biology. Overall, the results from the module preservation analysis, KEGG pathway enrichment, and hub gene identification converge to highlight the central role of mitotic regulators and cell cycle processes in NSCLC. The significant preservation of these modules across datasets and their association with key biological pathways emphasize the potential of targeting these regulators, such as *PLK1*, *CDK1*, and *CDC20*, for therapeutic intervention in NSCLC.

While the study successfully identifies key pathways and genes associated with non-small cell lung cancer (NSCLC), a significant limitation is the absence of experimental functional validation to confirm the biological significance of these findings. Without such validation, the roles of the identified genes and pathways remain speculative. Functional studies, such as gene knockdown or overexpression experiments, are crucial for substantiating the biological relevance of these genes in NSCLC. For instance, research has demonstrated the impact of specific genes on tumor progression through gene knockdown experiments, revealing their roles in cell proliferation and apoptosis [55]. Similarly, overexpression studies have shown how certain mutations, such as those in the *EGFR* gene, can promote aggressive tumor behavior [56]. These experiments provide direct evidence of how these genes influence cancer-related processes. Future research should focus on conducting these functional studies to validate the computational predictions and deepen the understanding of the molecular mechanisms driving NSCLC. This validation is essential to move from theoretical insights to practical applications in developing targeted therapies, as confirmed roles of these genes will guide the development of more effective treatment strategies.

#### 4.2.2. Signal Transduction

Non-small cell lung cancer (NSCLC) is frequently associated with mutations in *EGFR*, which affect up to 50% of East Asian populations and approximately 10% to 15% of cases in Western nations [57]. These mutations lead to uncontrolled cell growth and proliferation by activating downstream signaling pathways such as PI3K/AKT and MAPK [58]. The significant enrichment of the “Cell cycle” pathway observed in the study is closely linked to these signaling pathways. The PI3K/AKT/mTOR pathway is commonly altered in *EGFR*-mutant NSCLC. Research indicates that 67% of individuals with *EGFR* mutations exhibit basal activation of the AKT/mTOR axis [49]. Specific mutations within this pathway include: (1) *PIK3CA* mutations, which are present in 5–10% of *EGFR*-mutant NSCLC cases and confer resistance to *EGFR* TKIs, (2) loss of *PTEN*, which also results in resistance to *EGFR* inhibitors, and (3) *AKT1* mutations, although less frequent, are notable for their impact on treatment resistance [59]. The identification of hub genes related to the PI3K/AKT/mTOR pathway supports these findings. *AKT1*, an essential effector in the PI3K pathway, was found to be a critical hub gene within modules associated with cell cycle regulation and mitotic progression. Its overactivation is linked to poor prognosis in NSCLC, aligning with the results that show its significant role in the enriched “Cell cycle” pathway [50]. Targeting *AKT1* and other components of the *PI3K* pathway could potentially overcome *EGFR* TKI resistance, providing a valuable therapeutic strategy for NSCLC [60]. Moreover, BET inhibitors, which target *BRD4* and disrupt its interaction with acetylated histones, have shown promising anti-tumor effects in preclinical NSCLC models [61]. The integration of these findings into the broader context of the study highlights the importance of targeting key signaling pathways and genetic alterations in NSCLC for effective treatment strategies.

#### 4.2.3. Immune Response and Inflammation

The results from the gene co-expression network analysis and module preservation studies provide insights into the role of immune responses in NSCLC. Notably, the module preservation analysis identified several key modules related to immune function, including those associated with cell cycle regulation and mitotic progression. This association aligns with the preserved modules related to immune response identified in the analysis, suggesting a potential link between immune cell dynamics and the expression patterns of hub genes involved in cell cycle regulation.

The ability of *CD4+ T cells* to recognize neoantigens from mutations in oncogenes like *HER2* and *KRAS* further supports their role in targeting tumor-specific antigens [62,63]. This capability complements the findings from KEGG pathway analysis, which highlighted significant enrichment in pathways related to cell cycle and mitotic regulation. The convergence of these results emphasizes the interplay between tumor cell proliferation and immune responses, underscoring the potential for therapeutic strategies that leverage *CD4+ T cells* to enhance anti-tumor immunity [64] while targeting key cell cycle regulators such as *PLK1*, *CDK1*, and *CDC20*. Thus, integrating immune response insights with molecular findings provides a comprehensive view of NSCLC progression and potential therapeutic approaches. A further limitation of this study is the absence of an analysis correlating the identified gene expression patterns with clinical data, such as patient survival rates or treatment responses. Integrating clinical data could substantially enhance the clinical relevance of the findings by establishing links between the molecular features identified and patient outcomes. Such an analysis would provide a more comprehensive understanding of the clinical implications of the identified genes and pathways, potentially guiding more personalized treatment strategies. Future studies should aim to incorporate clinical data to validate and expand upon the current findings, thereby offering deeper insights into the prognostic and therapeutic potential of the identified molecular targets.

#### 4.2.4. The Influence of Estrogen on NSCLC Progression

It has been studied that estrogen receptors, in particular *ESR1*, contribute to the development of NSCLC. Research suggests that *ESR1* could control a number of signaling pathways, such as those involved in cell division, migration, and invasion, which influence the behavior of tumors. For example, bioinformatics investigations have demonstrated a correlation between increased expression of genes implicated in crucial signaling pathways, like *Wnt*/*β-Catenin* and *Notch*, which are known to play important roles in the development of NSCLC, and higher *ESR1* expression [65]. *ESR1* expression levels were shown to classify patients into groups with different prognoses in a study that analyzed gene expression data from patients with non-small cell lung cancer. More specifically, compared to patients with lower expression levels, those with increased *ESR1* expression showed superior overall survival rates [66]. This implies that *ESR1* may function as a prognostic marker in non-small cell lung cancer in addition to influencing tumor biology. The estrogen pathway plays a major role in lung adenocarcinoma by influencing a number of cancer hallmarks. By inducing pro-inflammatory cytokines, attracting regulatory T cells, and generating an immunosuppressive tumor microenvironment, estrogen regulates immunological responses. These processes support the migration, metastasis, and survival of cancer cells [67].

Interestingly, NSCLC cells can synthesize 17β-estradiol (E2) locally, similar to mechanisms observed in breast cancer. The two primary types of estrogen receptors via which E2 acts are ERα [68] and ERβ. ERα is expressed at lower levels in NSCLC, whereas ERβ is the more abundant protein. Research has demonstrated that ERβ mediates the proliferative effects of E2 in lung cancer cells, influencing the regulation of the cell cycle and promoting the formation of tumors via pathways such as *PI3K*/*Akt* and *MAPK*/*ERK* [69,70,71]. This receptor interaction increases the expression of genes associated with cell proliferation and survival, such as c-myc and cyclin D, and improves angiogenesis via vascular endothelial growth factor (VEGF) production [69,72].

**Figure 10:**
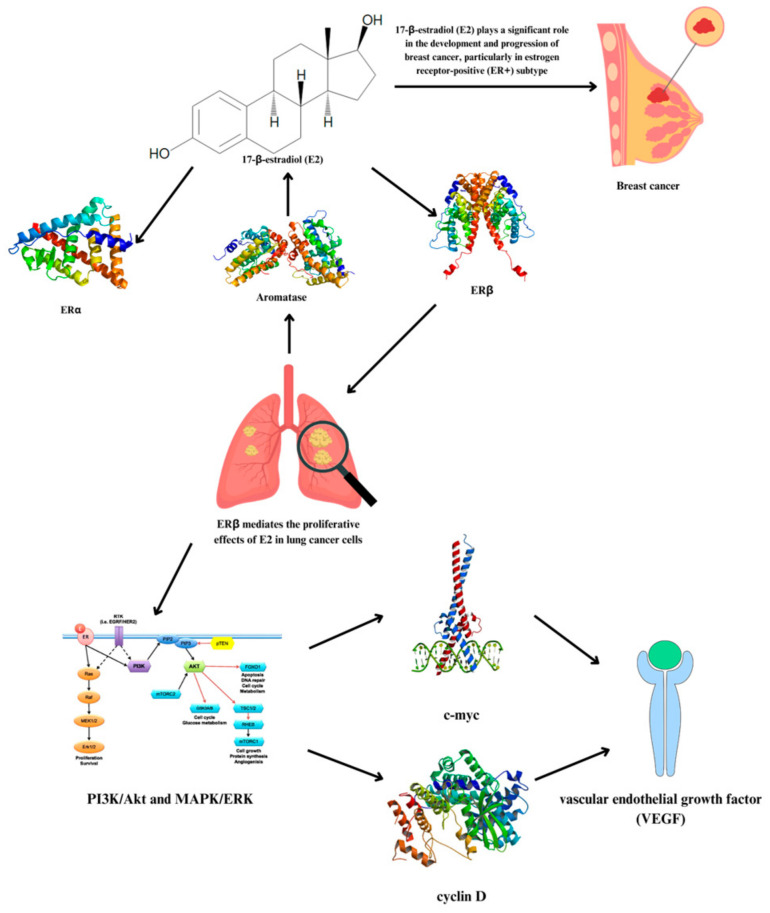
Local estrogen synthesis and estrogen receptor interaction in NSCLC and its potential metastasis to breast cancer.

On the other hand, aromatase, an enzyme that converts testosterone to estrogen, is substantially expressed in 44–86% of NSCLC tissues [72]. This is also linked to the biosynthesis of estrogen, particularly E2, which is a significant factor in the development and progression of breast cancer. Studies have shown that in postmenopausal breast cancer patients, the concentration of E2 in breast tissue can be significantly higher than in the plasma, suggesting that local estrogen production within tumors is a key factor in cancer progression [73,74]. A simple illustration of the process is shown in Figure 10.

### 4.3. Signature-Based Drug Repurposing

In the study of non-small cell lung cancer (NSCLC) progression, signature-based drug repurposing offers a promising approach by elucidating the complex interactions between various hub genes and their associated signaling pathways to identify potential therapeutic targets (Table 2). Preclinical studies have demonstrated that *GW-5074* has antitumor benefits, particularly when combined with other *RAF* inhibitors such as sorafenib. This combination has shown synergistic effects, significantly increasing cell death in renal cell carcinoma cell lines compared to each drug alone [75]. *GW-5074* inhibits *RAF*, disrupting the *EGFR–RAS–RAF–MEK–ERK* signaling cascade, which is crucial for cell proliferation and survival [76]. This suggests that GW-5074 could be effective against tumors with abnormal *EGFR* signaling due to mutations, such as those found in NSCLC. Additionally, one study discovered that GW5074 and sorafenib administration as part of a combination therapy considerably triggered necrotic death in various cancer cells in vivo and extended the survival of an animal disease model by dramatically suppressing primary and metastatic lesions. The study concluded that the co-administration of sorafenib with GW5074 showed anti-tumor effectiveness across a range of tumor types, in addition to a favorable safety profile [77]. Olomoucine may limit tumor growth by blocking both *CDK1* and its downstream effects on *CDC20*, particularly in cancers with dysregulated pathways. In the context of non-small cell lung cancer (NSCLC), combining *CDK* inhibition with the resulting impact on *CDC20* can be a strategic approach to limit the proliferation of cancer cells, potentially enhancing treatment effectiveness [78,79]. Pinocembrin treatment (50 μM) significantly reduced the number of migrating and invasive NSCLC cells by upregulating the protein level of E-cadherin and downregulating N-cadherin, vimentin and snail. This study shows that pinocembrin can prevent *STAT3* from being activated, which is linked to EMT, and thereby reduce NSCLC cells’ ability to migrate and invade [80]. Citalopram is a member of the selective serotonin reuptake inhibitor (SSRI) class of antidepressants, which is used to treat depression [81]. Interestingly, a systematic review highlighted that antidepressants, including citalopram, have been associated with anti-carcinogenic effects through various mechanisms such as inducing apoptosis, inhibiting cell proliferation, and modifying immune responses. This review emphasizes the need for more clinical studies to better understand the role of antidepressants in cancer treatment, including NSCLC [82]. Overall, these potential drugs—GW-5074, olomoucine, pinocembrin, and citalopram—offer promising new avenues for treating NSCLC by targeting upregulated and downregulated hub genes identified in the study. By addressing the signaling pathways and genetic alterations associated with NSCLC, these repurposed drugs could contribute to more effective therapeutic strategies. These findings offer valuable insights into potential new targets for drug discovery and contribute to a deeper understanding of the underlying biology of NSCLC. While the study successfully identifies key hub genes and potential drug repurposing candidates for NSCLC, a significant limitation is the absence of experimental validation for these findings. Without in vitro or in vivo validation, the functional relevance and therapeutic potential of the identified genes and drugs remain unconfirmed. This limitation underscores the necessity for future experimental studies to validate the computational predictions and ensure their applicability in clinical settings. Experimental validation would provide critical insights into the mechanistic roles of these genes and the efficacy of the proposed drug candidates, thereby strengthening the overall conclusions of the study.

## 5. Conclusions

In conclusion, this study underscores the pivotal role of Weighted Gene Co-expression Network Analysis (WGCNA) in elucidating the progression of non-small cell lung cancer (NSCLC) and identifying potential therapeutic targets. The analysis reveals a substantial gene co-expression overlap between NSCLC3 and NSCLC2, the reference dataset, with a significant correlation suggesting shared molecular characteristics across different disease stages. This highlights the robustness of the findings and their applicability across NSCLC stages. Enrichment analysis identifies highly preserved modules—Cyan, Yellow, Red, and Turquoise—as predominantly involved in cell division and inflammatory responses, aligning with the dysregulation of cell cycle processes critical to NSCLC pathology. Key hub genes, such as *PLK1*, *CDK1*, and *EGFR*, emerge as crucial regulators of tumor proliferation, signal transduction, and immune response. The KEGG pathway analysis further emphasizes the significance of pathways like “Cell cycle”, “Pathways in cancer”, and “Cell adhesion molecules” in NSCLC progression, reinforcing the importance of these pathways in tumor development. The study also highlights the influence of estrogen receptors, particularly *ESR1*, on NSCLC biology. Elevated *ESR1* expression correlates with improved survival outcomes, suggesting its utility as a prognostic marker. Additionally, the role of estrogen in regulating tumor biology through pathways such as *PI3K*/*Akt* and *MAPK*/*ERK* underscores its impact on cell proliferation and angiogenesis. The predominance of ERβ in NSCLC cells and its role in mediating estrogen effects further supports the need to consider hormonal factors in therapeutic strategies.

In terms of drug repurposing, the analysis identifies promising candidates such as GW-5074, which inhibits *RAF* and disrupts the *EGFR–RAS–RAF–MEK–ERK* signaling cascade, offering a potential strategy for targeting tumors with abnormal *EGFR* signaling. Preclinical studies have demonstrated that GW-5074 has antitumor benefits, particularly when combined with other *RAF* inhibitors such as sorafenib. This combination has shown synergistic effects, significantly increasing cell death in renal cell carcinoma cell lines compared to each drug alone. This suggests that GW-5074 could be effective against tumors with abnormal *EGFR* signaling due to mutations, such as those found in NSCLC. Additionally, one study discovered that GW-5074 and sorafenib administration as part of a combination therapy considerably triggered necrotic death in a variety of cancer cells in vivo and extended the survival of an animal disease model by dramatically suppressing primary and metastatic lesions. Additionally, olomoucine, by targeting *CDK1* and *CDC20*, aligns with observed dysregulation in mitotic pathways, suggesting a strategic approach to limit cancer cell proliferation. In the context of NSCLC, combining *CDK* inhibition with the resulting impact on *CDC20* can be a strategic approach to limit the proliferation of cancer cells, potentially enhancing treatment effectiveness. Furthermore, the study emphasizes the protective role of *CD4+* T cells in NSCLC, with higher *CD4+*/total T cell ratios associated with improved clinical outcomes. The ability of *CD4+* T cells to recognize tumor-specific antigens and their potential in immunotherapeutic strategies underscore the importance of integrating immune-based approaches into NSCLC treatment. Other potential drugs, such as pinocembrin, which reduces the number of migrating and invasive NSCLC cells by upregulating E-cadherin and downregulating N-cadherin, vimentin, and snail, show promise. This study shows that pinocembrin can prevent *STAT3* from being activated, which is linked to EMT, and thereby reduce NSCLC cells’ ability to migrate and invade. Additionally, citalopram, a member of the selective serotonin reuptake inhibitor (SSRI) class of antidepressants, has been associated with anti-carcinogenic effects through various mechanisms such as inducing apoptosis, inhibiting cell proliferation, and modifying immune responses. This review emphasizes the need for more clinical studies to better understand the role of antidepressants in cancer treatment, including NSCLC.

Overall, this comprehensive analysis enhances the understanding of NSCLC biology and provides valuable directions for future research and clinical applications. The integration of gene co-expression networks, pathway enrichment, hormonal influences, and drug repurposing offers a robust framework for developing targeted and effective treatment strategies.

## Figures and Tables

**Table 1:** GEO datasets summary.

Accession No.	GSE19804	GSE43580	GSE101929	GSE19804
Stage	1	2	3	4
Type	Expression profiling by array			
Platform	Affymetrix Human Genome U133 Plus 2.0 Array			
Source	Tumor lung tissue			
Organism	Homo sapiens			
No. of patients	35	26	11	12

**Table 2:** Top five drug candidates for the upregulated and downregulated hub genes for NSCLC.

Expression	Genes	Drug	Mechanism	Tau	FDR
Upregulated	*PLK1*, *EGFR*, *ESR1*, *CD4*, *CTNNB1*, *CREBBP*, *BRD4*, *AKT1*	L-745870	Dopamine receptor antagonist	−99.8	0.000188
		Pentolinium	Cholinergic receptor antagonist	−99	0.00189
		Gw-5074	Leucine-rich repeat kinase inhibitor, RAF inhibitor	−98.5	0.00481
		Pinocembrin	Cytochrome P450 inhibitor	−98	0.00208
		Bispherol-A	Synthetic estrogen	−97.5	0.000159
Downregulated	*CDK1*, *CDC20*, *RPS11*, *CCNA2*, *STAT1*, *SRSF1*, *BRCA1*, *CD20*, *CTNNB1*, *TLR4*, *ITGB2*, *PTPRC*, *RPS27A*, *HSP90AA1*	Clopidogrel	Purinergic receptor antagonist	−99.8	0.00396
		Rx-821002	Adrenergic receptor antagonist	−99.8	0.00416
		Fr-122047	Cyclooxygenase inhibitor	−99.6	0.00295
		Olomoucine	CDK indicator	−99.6	0.00596
		Citalopram	Selective serotonin reuptake inhibitor (SSRI)	−99.5	0.00596

## Data Availability

The gene microarray datasets used for the study are openly available in the NCBI Gene Expression Omnibus (GEO) database under the accession IDs GSE19804, GSE43580, and GSE101929 at https://www.ncbi.nlm.nih.gov/geo/ accessed on 25 July 2024.

## Appendix A

**Table A1:** Summary of hub genes and their protein functions from the GeneCards database.

Gene	Protein	Function	Modules
PLK1	Polo Like Kinase 1	Because its depletion in cancer cells significantly reduced cell growth and caused apoptosis, cancer therapy targets this protein.	Red/Yellow/Cyan
EGFR	Epidermal Growth Factor Receptor	Lung cancer is linked to mutations in this gene.	Royal Blue/Purple/White/Dark Turquoise
ESR1	Estrogen Receptor 1	This gene encodes a receptor that is important in osteoporosis, endometrial cancer, and breast cancer.	Royal Blue
CD4	CD4 Molecule	The protein stimulates or starts the early stage of T-cell activation and may be a key player in immune-mediated and viral disorders of the central nervous system that cause indirect neuronal damage.	Turquoise
CTNNB1	Catenin β 1	Colorectal cancer (CRC), pilomatrixoma (PTR), medulloblastoma (MDB), and ovarian cancer are all brought on by mutations in this gene.	Royal Blue/Dark Turquoise
CREBBP	CREB Binding Protein	This gene was first identified as a nuclear protein that binds to cAMP-response element-binding protein (CREB). Through the connection of chromatin remodeling to transcription factor recognition, it is now recognized to play important roles in embryonic development, growth control, and homeostasis.	Royal Blue/Dark Turquoise
BRD4	Bromodomain Containing 4	The chromosome 19 target of translocation t(15; 19)(q13; p13.1), which characterizes upper respiratory tract cancer in adolescents, has been linked to this gene.	Purple
CDK1	Cyclin Dependent Kinase 1	This protein’s phosphorylation and dephosphorylation play significant regulatory functions in the regulation of the cell cycle.	Red/Yellow/Cyan
CDC20	Cell Division Cycle 20	It appears that at various stages of the cell cycle, CDC20 functions as a regulatory protein through interactions with many other proteins.	Red/Yellow/Cyan
RPS11	Ribosomal Protein S11	This gene is distributed throughout the genome in several processed pseudogenes, as is common for genes encoding ribosomal proteins.	Royal Blue
CCNA2	Cyclin A2	This gene codes for a protein that is a member of the highly conserved cyclin family, which controls the cell cycle; adenocarcinoma and retinoblastoma are two diseases linked to CCNA2.	Yellow/Cyan
STAT1	Signal Transducer and Activator of Transcription 1	Receptor-associated kinases phosphorylate members of the STAT family in response to cytokines and growth factors. These members subsequently form homo- or heterodimers and translocate to the cell nucleus, where they function as transcription activators.	Dark Turquoise
SRSF1	Serine And Arginine Rich Splicing Factor 1	Depending on its contact partners and degree of phosphorylation, the encoded protein can either activate or suppress splicing.	White
BRCA1	BRCA1 DNA Repair Associated	Hereditary breast and ovarian cancers are now identified by the presence of germline BRCA1 mutations.	Red
TLR4	Toll Like Receptor 4	The Toll-like receptor (TLR) family of proteins, which includes the protein produced by this gene, is essential for pathogen identification and the induction of innate immunity.	Turquoise
ITGB2	Integrin Subunit β 2	Integrins are essential proteins found on the cell surface that are involved in both cell adhesion and signaling mediated by the cell surface.	Turquoise
PTPRC	Protein Tyrosine Phosphatase Receptor Type C	Cell growth, differentiation, mitosis, and oncogenic transformation are just a few of the biological processes that PTPs are known to influence as signaling molecules.	Turquoise
RPS27A	Ribosomal Protein S27a	It is produced from a precursor protein that is either a single ubiquitin coupled to an unrelated protein or a polyubiquitin chain.	Royal Blue
HSP90AA1	cluHeat Shock Protein 90 α Family Class A Member 1	This gene codes for an inducible molecular chaperone protein, which is a homodimer.	Purple/White

**Table A2:** Top KEGG pathways from highly preserved modules (z > 10).

Module	KEGG Term	Count	*p*-Value
Yellow	Metabolic pathways	242	2.03 × 10^−11^
	Pathways of neurodegeneration—multiple diseases	78	1.14 × 10^−4^
	Alzheimer disease	72	2.45 × 10^−6^
	Amyotrophic lateral sclerosis	66	2.18 × 10^−5^
	Cell cycle	59	9.41 × 10^−19^
White	Pathways in cancer	65	0.039962252
	Huntington disease	41	0.038434334
	Endocytosis	39	0.003801247
	Hepatocellular carcinoma	32	3.90 × 10^−4^
	Tight junction	32	3.90 × 10^−4^
Purple	Metabolic pathways	188	1.91 × 10^−5^
	Herpes simplex virus 1 infection	84	2.43 × 10^−7^
	Chemical carcinogenesis—reactive oxygen species	31	0.0235166
	Biosynthesis of cofactors	29	2.67 × 10^−4^
	Peroxisome	25	2.02 × 10^−7^
Turquoise	Pathways in cancer	85	2.89 × 10^−6^
	PI3K-Akt signaling pathway	63	4.31 × 10^−6^
	MAPK signaling pathway	52	3.76 × 10^−5^
	Rap1 signaling pathway	45	6.60 × 10^−7^
	Regulation of actin cytoskeleton	44	1.50 × 10^−5^
Royal Blue	Endocytosis	33	0.047955
	Cytoskeleton in muscle cells	31	0.044336
	Hepatocellular carcinoma	24	0.04709
	mTOR signaling pathway	23	0.03917
	Nucleocytoplasmic transport	22	1.84 × 10^−5^
Red	Metabolic pathways	202	2.29 × 10^−6^
	Pathways of neurodegeneration—multiple diseases	62	0.015828
	Cell cycle	58	6.81 × 10^−20^
	Alzheimer disease	54	0.006263
	Amyotrophic lateral sclerosis	51	0.008789
Cyan	Metabolic pathways	240	4.52 × 10^−9^
	Pathways of neurodegeneration—multiple diseases	84	1.69 × 10^−5^
	Alzheimer disease	77	3.46 × 10^−7^
	Amyotrophic lateral sclerosis	70	6.20 × 10^−6^
	Cell cycle	65	2.89 × 10^−22^
Dark Turquoise	Pathways in cancer	80	3.49 × 10^−4^
	MAPK signaling pathway	47	0.003029
	Shigellosis	40	0.004202
	Focal adhesion	39	1.49 × 10^−4^
	Cytoskeleton in muscle cells	39	0.002129
